# Rodent models of genetic epilepsy and its association with neurocognitive impairment- a systematic review

**DOI:** 10.3389/fphar.2025.1659569

**Published:** 2026-01-09

**Authors:** Renee Yan Ni Foo, Ian Juin Liang Chiew, Alina Arulsamy, Vanessa Lin Lin Lee

**Affiliations:** Neuroscience Research Strength, Jeffrey Cheah School of Medicine and Health Sciences, Monash University Malaysia, Selangor, Malaysia

**Keywords:** Animal model, cognitive impairment, genetic, epilepsy, rodent

## Abstract

Epilepsy is a neurological disorder affecting almost 50 million people worldwide, with genetic epilepsy (GE) representing a subset caused by specific gene mutations. While cognitive deficits are frequently reported in epilepsy, the contribution of GE itself remains poorly defined. We conducted a systematic review to evaluate the cognitive and behavioral phenotypes in rodent models of GE, focusing on cognition as the primary outcome and behavior as secondary. Literature searches of PubMed, Ovid MEDLINE, and Scopus identified 16 eligible studies in accordance with Preferred Reporting Items for Systematic Reviews and Meta-Analyses (PRISMA) guidelines. Across models, rodents with GE commonly exhibited impairments in the neurocognitive and behavioral paradigms. Mutant rodent models were exhibit poorer memory and learning abilities, alongside behavioral abnormalities such as autism spectrum disorder (ASD)-like phenotype, anxiety, and depression. However, the severity and domains of impairment varied across mutations, strains, and developmental stages, reflecting the heterogeneity of GE. Our findings highlight both seizure-driven and gene-driven mechanisms of cognitive impairment and underscore the need for syndrome-specific investigations. Overall, rodent models provide valuable insights into the cognitive comorbidities of GE, but future research requires improved methodological rigor and broader use of complementary models to clarify underlying mechanisms and guide targeted interventions.

## 1 Introduction

Rapid genomic technological breakthroughs in recent years have made genetics an increasingly important field of study. The study of genes offers insights into fundamental processes from birth to death. It also provides information about disease etiology and potential therapies, enabling more effective use of existing treatments to address prevalent health conditions because of the crucial role of genetic activity in all biological processes (Vadlamudi et al., 2014).

Epilepsy is clinically defined as a chronic neurological disorder characterized by recurrent episodes of unprovoked seizures and is clinically recognized to be accompanied with other cognitive, psychological, neurobiological and social complications (Fisher et al., 2014). Epileptic seizures are caused by synchronous and excessive electrical brain discharges, triggered by abnormally functioning voltage-gated and ligand-gated ion channels that result in electrically hyperactive neurons (Fisher et al., 2014; Novak et al., 2022). As a result, epileptic seizures manifest as transient neurological signs and symptoms (Fisher et al., 2014). The ILAE classifies seizures into generalized, focal, or unknown onset (Fisher et al., 2017). The lifetime prevalence of epilepsy is 7.6 per 1,000 persons and the annual cumulative incidence is 67.77 per 100,000 persons, with prevalence higher in men than in women (Fisher et al., 2017; Novak et al., 2022).

According to the International League Against Epilepsy (ILAE) classification, epilepsies are categorized into six etiologic groups: structural, genetic, infectious, metabolic, immune, and unknown. The term “Genetic Epilepsy” used in this review corresponds to the group of *Genetic Generalized Epilepsies* (*GGEs*), which include syndromes such as Childhood Absence Epilepsy, Juvenile Absence Epilepsy, and Juvenile Myoclonic Epilepsy. These conditions are characterized by a presumed genetic basis, although the specific causative mutations are not always identified (Scheffer et al., 2017). A review identified 84 epilepsy genes, defined as gene mutations that cause either pure epilepsies or syndromes in which epilepsy is the presenting symptom (Wang et al., 2017). GEs often follow a complex inheritance pattern which may result from familial inheritance or sporadic single or multiple gene mutations, with or without an environmental influence, leading to the development of specific epileptic phenotypes (Scheffer et al., 2017).

Channelopathies, caused by mutations in genes coding ion channels or their accessory subunits, are rare but predominant causes of GE (Steinlein, 2008). Voltage-gated ion channels, involved in the generation and propagation of action potentials (e.g., sodium, potassium, or chloride channels, or ligand-gated ion channels, which mediate synaptic conduction (e.g., acetylcholine or GABA receptors) are among the classes of ion channels implicated in channelopathies (Steinlein, 2004). The *SCN1A* gene, which encodes one of nine voltage-gated sodium channels essential for neurological function, is the most common pathogenic gene associated with Dravet syndrome (DS) (Ding et al., 2021; Wang et al., 2017). Loss-of-function mutations in the *SCN1A* accounts for approximately 80% of DS cases (Ding et al., 2021). Mutations in other epilepsy related genes, such as *GABRG2*, *GABRB3*, *CACNA1H*, and *GABRA1*, have been implicated in childhood absence epilepsy (CAE) (Wang et al., 2017). PCDH19 clustering epilepsy is a rare monogenic epilepsy syndrome caused by a loss-of-function mutation of the protocadherin-19 (*PCDH19*) gene, which encodes a calcium-dependent adhesion molecule involved in cell-cell adhesion and synaptic communication (Moncayo et al., 2022). This syndrome follows a unique X-linked inheritance as it primarily affects heterozygous females, while hemizygous males are usually asymptomatic (Moncayo et al., 2022; Samanta, 2020). Random X inactivation in females produces somatic mosaicism of cells with and without PCDH19 protein, leading to dysfunctional cellular interference (Samanta, 2020).

There is more to epilepsy than just seizures. Individuals with epilepsy are often burdened by comorbidities that are more debilitating than seizures themselves. Epilepsy is commonly associated with cognitive dysfunction, such as learning impairment, memory deficits, and intellectual disability, as well as neuropsychiatric comorbidities including anxiety disorders, depressive disorders, and autism spectrum disorder (ASD) (Scheffer et al., 2017). These comorbidities severely affect the quality of life of epileptic patients (Ding et al., 2021). Clinical and narrative literature has suggested that uncontrolled epilepsy may be associated with functional and structural brain alterations that manifest as cognitive deficits (Novak et al., 2022). However, there remains a knowledge gap regarding the direct effects of GE itself, due to its inherent genetic mutations or other coexisting factors, on cognition in patients with minimal seizure history. An alternative theory proposes that both seizure and cognitive comorbidities stem from disrupted neural networks caused by underlying pathogenic etiology (Khalife et al., 2022). Evidence also suggests that cognitive impairment is evident before the onset of seizure in newly diagnosed children, indicating that cognitive deficits may be result from the same underlying dysregulation that causes seizures, rather than seizures themselves (Khalife et al., 2022). Moreover, in DS, the severity and frequency of seizures have been reported to show no direct correlation with the severity of cognitive dysfunction (Lenck-Santini and Scott, 2015). Collectively, these findings suggest that the underlying pathology in GE plays a key role in the cognitive decline, independent of seizure-related mechanisms.

Animal models are essential for studying epilepsy because it is ethically unfeasible to induce epileptogenesis and ictogenesis in human trials. Due to their genetic and morphological similarities to humans, rodents have long been the preferred species in epilepsy research, especially in studies involving genetic modification and targeted mutations. For this review, knock-out and knock-in rodent models are particularly valuable, as they allow targeted manipulation of specific gene loci and often recapitulate phenotypic traits seen in human (Simmons, 2008). Several rodent strains have been proposed as models of GE including EL mice, genetically epilepsy-prone rats (GEPRs) and absence epilepsy strains such as Wistar Albino Glaxo from Rijswijk (WAG/Rij) and Genetic Absence Epileptic Rats from Strasbourg (GAERS), which display recurrent spontaneous seizures accompanied by cognitive-behavioral abnormalities (van Luijtelaar, 2011). Both WAG/Rij and GAERS are well-established models of human generalized absence epilepsy (GAE), faithfully replicating recurrent absence seizures characterized by reduced responsiveness and synchronous spike-wave discharges (SWD), mirroring typical features observed in human features (Coenen and Van Luijtelaar, 2003; Marescaux et al., 1992).

Although it is essential to develop animal models that capture the full clinical features of epilepsy, particularly cognitive deficits, it remains a major challenges. For this review, cognition’ refers to a set of higher-order mental processes encompassing learning, memory, attention, and executive function, as operationalized through validated behavioral tasks in rodent models. The term “neurocognitive” is used to emphasize the neural mechanisms underlying these cognitive processes. Therefore, the present study aims to systematically review the literature to investigate the effects of GE on cognition in rodent models. This work will improve our understanding of epileptogenic mechanisms and their cognitive-behavioral consequences, and help identify potential targets for novel therapeutic interventions to improve cognitive outcomes in patients with epilepsy. Understanding the cognitive deficits associated with genetic epilepsies provides insight into the underlying neuropharmacological mechanisms that link genetic mutations to altered neuronal excitability, synaptic plasticity, and neurotransmitter regulation. Such knowledge is essential for the development of targeted therapeutic interventions aimed at mitigating both seizure activity and comorbid cognitive impairments.

## 2 Methodology

### 2.1 Data source and search strategy

A literature search was performed using three electronic databases, Scopus, PubMed, and Ovid MEDLINE, covering publications from database inception until 11th March 2024. The search terms applied were “Animal model*” AND “Cognit*” AND “Genetic epilep*”.

### 2.2 Study eligibility criteria

Only original studies written in English were included in this study. The inclusion criteria comprised all original studies on rodent models that examined the effect of GE on cognition. Non-original articles (i.e., systematic or literature reviews, book chapters, abstracts, etc.) and studies that did not meet the inclusion criteria were excluded during screening. Non-original studies were omitted due to insufficient datafor comparison and evaluation. Studies were included if they (i) employed rodent models with confirmed genetic or transgenic modifications linked to epilepsy phenotypes, (ii) assessed cognitive functions using validated behavioral paradigms (e.g., Morris Water Maze, Barnes Maze, Fear Conditioning, Novel Object Recognition), and (iii) reported quantitative outcomes. Studies involving solely pharmacological or environmental induction without genetic manipulation were excluded.

### 2.3 Data extraction and analysis

**FIGURE 1:**
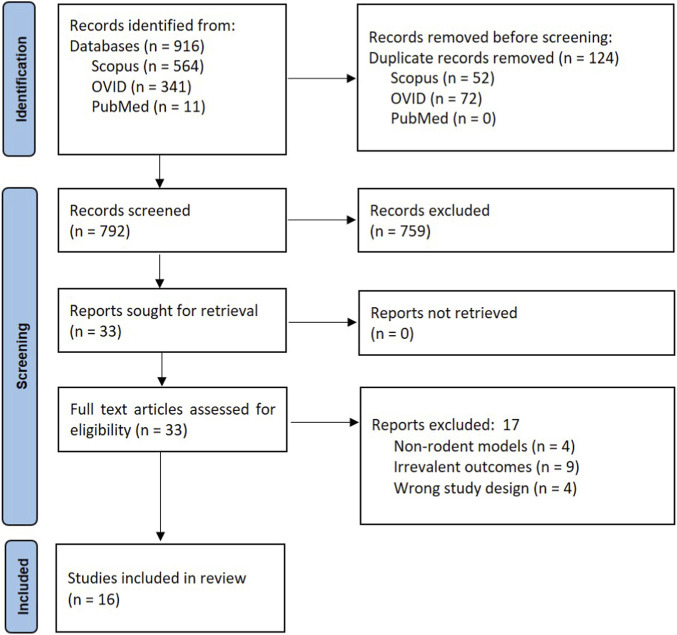
Flow diagram of literature search strategy and study selection process based on the Preferred Reporting Items for Systematic reviews and Meta-Analyses (PRISMA) Guidelines.

Two authors were involved in extracting the data. The studies obtained through database search were exported into Covidence, a tool used to streamline systematic reviews. Covidence enabled automatic removal of duplicated articles, and the remaining articles were initially reviewed through title and abstract screening. Following the elimination of studies that failed to meet the inclusion criteria, full-text articles were evaluated for eligibility (Figure 1).

### 2.4 Quality assessment

The methodological quality of included studies was assessed using the Systematic Review Centre for Laboratory animal Experimentation (SYRCLE) Risk of Bias (RoB) tool. This tool consists of 10 domains covering selection, performance, detection, attrition, reporting, and other sources of bias. Each domain was rated as “Yes” (low risk of bias), “No” (high risk of bias), or “Unclear” (insufficient information). Two authors independently assessed each article, with discrepancies resolved through discussion.

## 3 Results

### 3.1 Selection of studies

A search of the three databases using the keywords mentioned in the methodology yielded 916 articles, followed by the removal of 124 duplicates. Titles and abstracts of the remaining 792 articles were screened, and 759 articles were excluded for not meeting the inclusion criteria. A total of 33 articles were included for full-text screening, of which 17 articles were omitted for the following reasons: (a) nine had wrong outcomes, (b) four involved non-rodent model, and (c) four had inappropriate study designs (Figure 1). Ultimately, 16 eligible articles were extracted and included for discussion in this systematic review, as compiled in Table 1.

**TABLE 1:** Tabular representation of the rodent models of genetic epilepsy as well as the cognitive and neuropsychiatric and behavioural outcomes of the studies.

Author/References	Animal model	Results of cognitive outcomes	Results of neuropsychiatric and behavioural impairment
Aguilar et al. (2018)	Male and female genetically epilepsy-prone rats (GEPR-3s) and SD rats	Novel object recognition test • GEPR-3s demonstrated poor novel object preference, suggesting long-term memory impairment	Open field test • In both SD and GEPR-3 strains, males explored the arena lesser females • Female GEPR-3s explored the centre of the arena significantly less as compared to female SD rats, suggesting an increase in anxiety-related behaviours Elevated plus maze • Female GEPRs showed fewer head dips into the open arms relative to female SD rats • GEPR-3s showed lower number of head pokes into the open arms relative to control rats • GEPR-3s exhibited more protected stretch-attend posture than SD rats in the closed arm, suggesting anxiety-related behaviour Light-dark transition test • GEPR-3s displayed reduced time spent in the light compartment relative to control SD rats and is worse in males than females, revealing increased anxiety Looming threat test • During the post-stimulus period, GEPR-3s showed a significant increase in freezing as compared to SD rats, indicating increased anxiety Sucrose preference test • GEPR-3s showed a significantly lower sucrose preference and consuming less sucrose relative to SD rats, likely displaying depressive behaviour
Marks et al. (2016b)	Male and female genetic absence epilepsy rats from Strasbourg (GAERS) and non-epileptic strain (NEC)	Pavlovian fear conditioning test • GAERS displayed heightened fear-related behaviour and increased associative memory functions relative to aversive stimuli	Elevated plus maze • GAERS in prepubertal age and young adulthood displayed heightened anxiety behaviour Open field test • GAERS displayed altered explorative behaviour but no anxiety-related behaviour
Marks et al. (2019)	Male and female genetic absence epilepsy rats from Strasbourg (GAERS) and non-epileptic strain (NEC)	Pavlovian fear conditioning test • GAERS displayed heightened fear-related behaviour and increased associative memory functions relative to aversive stimuli	​
Marks et al. (2016a)	Male and female genetic absence epilepsy rats from Strasbourg (GAERS) and non-epileptic strain (NEC)	Crossmodal object recognition test • GAERS demonstrated impaired recognition memory in non-aversive settings/tasks	​
Russo et al. (2013)	Male Wistar Albino Glaxo from Rijswijk (WAG/Rij) rats Intervention: Treatment with aripiprazole (APZ)	Morris water maze • The authors reported that APZ has shown to effectively improve learning and adaptive memory functions as well as recall memory in WAG/Rij rat strains	Sucrose consumption test • APZ treatment, at 0.3 and 1 mg/kg, significantly increased sucrose intake in WAG/Rij rats, indicating reduction in depressive behaviour Forced Swimming test • APZ at 1 and 3 mg/kg, significantly lowered immobility time by 22% and 34% respectively, indicating an increase in hedonic capacity Elevated plus maze • APZ was significantly effective 1 mg/kg in increasing the time spent in the open arm and reducing the time spent in the closed arm and in the centre, suggesting a reduction in anxiety-related behaviours Open field test • APZ significantly increase the number of rearing and grooming behaviour at all doses and the number of centre entries at the two highest doses, indicating reduced anxiety-related behaviours
Jafarian et al. (2015)	Male Wistar Albino Glaxo from Rijswijk (WAG/Rij) rats and Wistar rats	Passive avoidance test • WAG/Rij rats demonstrated age-dependent learning and memory deficits	​
Cwetsch et al. (2022)	Male and female Sprague-Dawley (SD) rats Intervention: *In utero* electroporation with *Pcdh19* downregulated shRNA to express focal mosaicism of PCDH19 downregulated cells and wild-type cells	Novel object recognition test • *Pcdh19* shRNA-electroporated SD rats demonstrated poor novelty-discrimination between familiar and novel objects, signifying impaired long-term memory functions Contextual fear-conditioning test • *Pcdh19* shRNA-transfected rats displayed impairments in associative memory	Ultrasonic vocalisation test • *Pcdh19* shRNA-electroporated pup rats vocalized less than control littermates, indicating socio-behavioural deficits Huddling test • *Pcdh19* mutant pups demonstrated more pronounced isolative behaviour relative to their control littermates Three-chamber test • *Pcdh19* shRNA-transfected rats displayed a significantly decreased “sociability index,” indicating that the social-behavioural impairments persisted into adulthood Hot plate tests • Rats subjected to *Pcdh19* shRNA electroporation exhibited a notably reduced latency response to an acute thermal stimulus relative to control rats, suggesting sensory hypersensitivity
Bender et al. (2016)	Male, adult Sprague-Dawley (SD) rats Intervention: Treatment with shRNA sequences targeting rat *Scn1a* gene	Morris water maze • *Scn1a*-treated rats showed intact spatial reference memory, however, short-term memory impairment was reported T maze rewarded alternation • *Scn1a* mutant rats displayed worse performance than control rats suggesting significant working memory deficit	Open field test • *Scn1a* mutant rats spent lesser time exploring the centre of the arena compared to control rats, suggesting an increased anxiety-like behaviour
Dutton et al. (2017)	Female and male C57BL/6J background mice Intervention: *Scn1a* R1648H mutation (RH line)	Three-chambered social interaction test • APFE *RH/+* displayed social recognition and memory deficits Novel object recognition test • APFE *RH/+* showed recognition memory deficits	Open field test • APFE *RH/+* mice demonstrated hyperactivity but normal anxiety levels Novel cage test and forced swim test • *RH/+* mice reported normal exploratory behaviours and no exhibition of depressive moods
Phillips et al. (2014)	Female and male DBA/2J or C57BL/6J background mice Intervention: SWD-associated transcriptional downregulation of hippocampal HCN1 (R43Q)	Morris water maze • R43Q mouse from DBA/2J background displayed spatial learning and memory deficit • The seizure-resistant strain with similar mutations, R43Q mouse from C57BL/6J background, displayed no memory impairments	​
Qu et al. (2020)	Female and male C57BL/6J background mice Intervention: Knock-in *Gabrb3* ^*+/D120N*^ mutation	Barnes maze test • Young and adult KI mice exhibited both spatial learning and spatial memory deficits	Locomotor activity test • Young and adult KI mice showed hyperactivity elevated plus maze • KI mice displayed mild anxiety that worsens with age Three-chamber Socialization test • Young KI mice showed a significant reduction in overall exploratory behaviour but adult KI mice only displayed reduced exploratory behaviour • Young KI mice had reduced socialization, which evolved into completely abnormal socialization during adulthood
Nwosu et al. (2023)	Female and male C57BL/6J background mice Intervention: Knock-in *Gabrb3* ^*+/N328D*^ mutation	Barnes maze test • *Gabrb3* ^*+/N328D*^ mice reported spatial memory and spatial learning deficits	Elevated-zero maze • *Gabrb3* ^*+/N328D*^ mice did not exhibit anxiety but a deficit in exploratory ability
Lena and Mantegazza (2019)	Male C57BL/6N background mice Intervention: Knock-out Na_V_1.2 haploinsufficiency in the heterozygous *Scn2a* gene	Y Maze • Young *Scn2a* ± mice displayed mild spatial working memory impairments • No significant difference was seen in adult mice Novel object recognition task and barnes maze test • Young *Scn2a* ± mice exhibit impairment in recognition memory and spatial working memory, while adult heterozygous mice had intact spatial working, long-term memory, and recognition memory, with only a tendency of slower spatial learning	Ultrasonic vocalizations • Young and adult *Scn2a* ± mice displayed deficits in social communication Self-grooming behaviour and marble burying test • Young *Scn2a* ± displayed stereotyped and repetitive behaviours which were not seen in adult *Scn2a* ± mice Tail suspension test • Young *Scn2a* ± mice are less resigned than adult mutants Open-field test and elevated plus maze • Young Scn2a ± mice showed less anxiety compared to adult *SCN2A* ± mice
Qu et al. (2023)	Female and male C57BL/6N background mice Intervention: Knock-in *Gabrb3* ^*+/N110D*^ mutation	Barnes maze test • *Gabrb3* ^*+/N110D*^ mice displayed slower acquisition in spatial learning and impaired spatial memory	Three-chamber Socialization test • KI mice exhibited impaired sociability Open field test and elevated-zero maze • KI mice reported increased anxiety and hyperactivity
Salgueiro-Pereira et al. (2019)	Female and male hybrid mixed 129P2/OlaHsd x C57BL/6J background mice Intervention: Heterozygous knock-in R1648H Na_v_1.1 (*Scn1a*) mutation (*Scn1aRH/+*)	Morris water maze • *Scn1a* ^*RH/+*^-SIH and *Scn1a* ^*RH/+*^-SIF showed impaired spatial memory, spatial learning, and long-term memory	Open field test • *Scn1a* ^*RH/+*^-SIH and *Scn1a* ^*RH/+*^-SIF mice showed anxiety and stereotyped behaviour Three-chamber test • *Scn1a*-SIH and *Scn1a* ^*RH/+*^-SIF mice both displayed impaired sociability and social novelty skills
Gheyara et al. (2014)	Female and male C3HeB/FeJ x C57BL/6J background mice Intervention: Knock-in truncation mutation in the *Scn1a* gene (R1407X)	Barnes maze test • *Scn1a* ^*RX/+*^ mice showed long-term memory deficits and impaired spatial learning • Tau ablation brought latency, distance, and strategy measures in *Scn1a* ^*RX/+*^ mice to control levels Contextual fear conditioning • *Scn1a* ^*RX/+*^ mice had deficits in associative memory and learning • Tau ablation ameliorated deficits in associative learning and memory in *Scn1a* ^*RX/+*^ mice	Open field test • *Scn1a* ^*RX/+*^ mice showed hyperactivity and increased anxiety • Tau ablation ameliorated the hyperactivity of *Scn1a* ^*RX/+*^ mice, but not reaching statistical significance

### 3.2 Animal models

The eligible studies involved rodent models, including both mice and rat models. Eight studies used mice, while another eight used rat, contributing to the total of sixteen eligible studies. Among the eight rat studies, four different strains were used. One study used the GEPR-3s strain (Aguilar et al., 2018), three studies used the GAERS strain (Marks et al., 2016a; Marks et al., 2016b; Marks et al., 2019), two employed the WAG/Rij strain (Jafarian et al., 2015; Russo et al., 2013), and two studies used genetically modified Sprague-Dawley (SD) rats with shRNA encoding downregulation of the *Pcdh19* gene (Cwetsch et al., 2022) or the *Scn1a* gene (Bender et al., 2016). The control rats used for comparison were of the same background: transgenic studies used SD rats with or without control shRNA sequences (Bender et al., 2016; Cwetsch et al., 2022); GEPR-3s studies used SD control rats (Aguilar et al., 2018); WAG/Rij rat studies employed either Wistar rats or WAG/Rij rats injected with vehicle; and GAERS studies used the non-epileptic control (NEC) strain (Marks et al., 2016a; Marks et al., 2016b; Marks et al., 2019).

In the eight mouse studies, transgenic models were used, representing four different strains. Five studies used the C57BL/6J strain, targeting mutations in genes such as *Scn1a*, *Scn2a*, and the GABA_A_ receptor β3 Subunit (Dutton et al., 2017; Lena and Mantegazza, 2019; Nwosu et al., 2023; Phillips et al., 2014; Qu et al., 2020). Among these, one study employed both C57BL/6J strain and DBA/2J strains, encoding for SWD-related transcriptional downregulation of the hippocampal HCN1 channel gene, resulting in the R43Q mutation (Phillips et al., 2014). One study used the C57BL/6N strain with a *Gabrb3* ^*+/N110D*^ gene knock-in (Qu et al., 2023). Two studies used mice with mixed backgrounds: one used 129P2/OlaHsd mixed C57BL/6J hybrids carrying an *Scn1a* mutation (Salgueiro-Pereira et al., 2019), while the other study used C3HeB/FeJ mixed C57BL/6J hybrids with a truncation mutation in *Scn1a* gene (Gheyara et al., 2014). Notably, four of the sixteen studies evaluated only male rodents (Lena and Mantegazza, 2019), while the remaining studies included both sexes to investigate the sex difference in cognitive function (Dutton et al., 2017; Gheyara et al., 2014; Nwosu et al., 2023; Phillips et al., 2014; Qu et al., 2020; Qu et al., 2023; Salgueiro-Pereira et al., 2019).

### 3.3 Methods of cognitive assessment

Cognition encompasses both basic and complex mental functions. Basic functions include attention, sensation, and perception, while the complex processes involve learning, memory, decision-making, and problem-solving. As the primary aim of this systematic review was to assess cognitive impairment in rodent models with GE, a variety of tests were used to assess memory functions in genetically mutated strains.

The cognitive assessments employed can be broadly divided into two categories; (1) tests of associative long-term memory in response to aversive stimuli, such as fear conditioning; (2) tests of learning and memory functions without aversive stimuli, including short-term recall, working memory, long-term recall, reference memory, adaptive memory, and recognition memory.

The Barnes Maze Test (BMT) was frequently used to assess spatial memory, spatial learning, recall memory and adaptive memory. Other tasks, including the Morris Water Maze (MWM), 8-arm radial maze, T maze rewarded alternation test, and Y-maze test evaluated similar parameters, with addition focus on long-term memory in rodents with GE. Social recognition memory was assessed using the three-chambered social interaction test, which determined whether mutant rodents spent more time with novel mice compared to familiar conspecifics. Other commonly used assessments included the novel object recognition test (NORT) and contextual fear conditioning (CFC), which evaluated recognition memory, long-term recall memory, and associative learning and memory, respectively. Associative memory for aversive stimuli was also measured with fear conditioning paradigms (low- and high-intensity) and the passive avoidance test. In some studies, recognition memory was further assessed using tactile, visual, and cross-modal object recognition (CMOR) test.

Because some GEs are associated with ASD-like phenotype, this review also evaluated the complex interplay between GE and ASD as a secondary outcome. All studies assessed behavioral and neuropsychiatric comorbidities relevant to ASD, such as anxiety, depression, intellectual disability, and hyperactivity. The most common tests for anxiety were open field test (OFT) and elevated plus maze (EPM) which also evaluated locomotion deficits. Other tasks used for similar purposes included the three-chamber test, locomotor activity chambers, looming threat test, and dark light test. Depression-related behaviors were assessed using tail suspension test (TST), sucrose consumption test (SCT), and forced swim test (FST). Repetitive behaviors, a hallmark of ASD, were examined using self-grooming and marble burying tests. Social interaction deficits were evaluated using ultrasonic vocalizations (USV), huddling test, reciprocal social interaction test, and the three-chambered social interaction test. Finally, sensory alterations were assessed using the hot plate test.

### 3.4 Primary outcome

#### 3.4.1 Short-term memory

Spatial learning and memory were impaired in *Scn1a* mutant rats as assessed by the Morris Water Maze (MWM). MWM evaluates hippocampal-dependent spatial learning by measuring the ability of rodents to locate a hidden escape platform in a pool of water. A study by Bender et al. concluded that in this paradigm, *Scn1a* mutant rats showed significantly poorer path efficiency in locating the platform compared to controls. Additionally, *Scn1a*-treated rats also crossed over the target quadrant significantly less compared to the control group (Bender et al., 2016). The T maze rewarded alternation test concluded that the genetically modified SD rats with expression of *Scn1a* gene mutation revealed significantly poorer performance with a choice accuracy of approximately 68.8% compared to 80.6% in control rats. During variable delay trials, a more pronounced performance decline was observed in the *Scn1a* gene mutated rats with a choice accuracy of merely 56.8% in the longest delay period whereas a performance improvement was observed in the control group (Bender et al., 2016). The results from both tests concluded that SD rats with mutated *Scn1a* gene displayed significant spatial memory deficit compared to the control rats.

Short-term spatial learning deficits were also evident in *Scn1a* mutant mice when tested in the BMT. BMT is a dry-land spatial learning task in which rodents use visual cues to locate a hidden escape hole on a circular platform. In this paradigm, *Scn1a* mutants exhibited longer escape latencies and committed more errors before finding the target hole compared with controls. Researchers interpreted these findings as evidence of impaired short-term spatial learning (Gheyara et al., 2014; Nwosu et al., 2023; Qu et al., 2020; Qu et al., 2023). These deficits were shown to be present in test groups consisting of both younger (49-day-old) and older (200-day-old) *Gabrb3* ^*+/D120N*^ mutant mice (Qu et al., 2020). Similarly, *Scn1a* mutant mice showed a significantly lower performance by not reaching the criterion of making more than 75 percent correct choices to reach the escape platform during the last two consecutive days of training as compared to their controls in the MWM (Salgueiro-Pereira et al., 2019).

However, utilization of the Y-maze test in *Scn2a* knock-in mice showed that despite young *Scn2a* mutants displayed only a borderline significant decrease in the percentage of spontaneous alterations compared to wild-type mice in the test, no significant difference was found in adult *Scn2a* knock-in mice (Lena and Mantegazza, 2019). Furthermore, mice with R1648H mutation of *Scn1a* gene will only present with cognitive and behavioral deficits if they have been previously exposed to febrile seizures induced by hyperthermia (SIH) or by flurothyl (SIF) (Salgueiro-Pereira et al., 2019). Interestingly, though R43Q mice were shown to exhibit spatial memory and spatial learning impairments, their seizure-resistant strain which expresses the same mutation showed contrasting results (Phillips et al., 2014).

#### 3.4.2 Long-term memory

The Novel Object Recognition Test (NORT) is commonly used to assess long-term memory in rodents, as it leverages their innate preference for exploring novel objects over familiar ones. One study revealed that in the NORT, rats electroporated with *Pcdh19* shRNA displayed poor novelty-discrimination abilities spending similar amounts of time with novel and familiar objects (Cwetsch et al., 2022). Consistent with this, Aguilar et al. (2018) reported that Genetic Epilepsy-Prone Rats (GEPR-3s) showed poor novel-object preference, performing at chance levels, whereas Sprague–Dawley control rats displayed the expected significant preference for novelty (Aguilar et al., 2018).

Similarly, Salgueiro-Pereira et al. (2019) found that *Scn1a* mutant mice in SIH and SIF groups exhibited deficits in memory performance, spending significantly less time in the target quadrant during the Morris Water Maze probe test and failing to discriminate between adjacent and target quadrants. This pattern suggests impairments not only in spatial learning but also in long-term memory (Salgueiro-Pereira et al., 2019). Together, these findings demonstrate convergent evidence from multiple models that genetic epilepsies are associated with impairments in long-term recognition memory, as revealed by NORT, and in spatial memory, as assessed by MWM.

Long-term spatial memory was also impaired in *Scn1a* mutant rats when assessed with the Morris Water Maze (MWM). In this version of the MWM, rodents undergo repeated training sessions across multiple days, which allows evaluation of long-term memory retention. In this version of the MWM, rodents undergo repeated training sessions across multiple days, which allows evaluation of long-term memory retention. It was discovered that the untreated WAG/Rij group required a longer time and crossed more quadrants before locating the platform compared to the treatment group during day 6 of testing (Russo et al., 2013), which yielded similar results among *Scn1a* and *Gabrb3* ^*+/N110D*^ mutant mice (Gheyara et al., 2014; Qu et al., 2023; Salgueiro-Pereira et al., 2019).

However, the MWM test conducted by Bender et al. showed that there was no significant difference observed between *Scn1a* mutant rats and control rats in terms of the latency to locate the platform or the duration spent in the target quadrant which signifies that spatial reference memory remains intact in these rats (Bender et al., 2016).

#### 3.4.3 Recognition memory

Recognition memory, which reflects the ability to discriminate between familiar and novel stimuli, was primarily assessed using variations of the Novel Object Recognition Test (NORT) and cross-modal object recognition tasks.

In a study by Dutton et al. (2017), *Scn1a* mutant mice exposed to either a prolonged febrile event (PFE) or an acute/prolonged febrile event (APFE) exhibited divergent outcome. While both wild-type and PFE mutants were able to distinguish between familiar and novel objects, only the APFE mutants spent equal time with both objects (Dutton et al., 2017).

Lena and Mantegazza (2019) reported an age-dependent effect in *Scn2a* mutant mice: recognition memory deficits were observed in young mutants but did not persist in adulthood (Lena and Mantegazza, 2019).

In the drug-naive trials, NEC rats outperformed GAERS in the visual and tactile recognition test although a significant strain difference was not appreciated. Throughout both tests, both strains showed statistically significant recognition memory above chance level. In the CMOR test, the NEC strain displayed significant novel object exploration during all time periods of the test while the GAERS strain did not (Marks et al., 2016a). These results show that GAERS exhibit impaired recognition memory in non-aversive settings.

Collectively, these studies indicate that recognition memory impairments are a recurring phenotype across multiple genetic epilepsy models. However, the expression of deficits may be influenced by developmental stage, seizure history, and task demands, underscoring the complexity of cognitive outcomes in GE.

#### 3.4.4 Associative memory

Associative memory, which reflects the ability to form and recall stimulus–response associations, was commonly assessed using passive avoidance paradigms and fear conditioning tasks.

In a passive avoidance test, 6-month-old WAG/Rij rats showed significantly reduced step-through latency response into the dark compartment at 1-week and 1-month post-acquisition, compared to Wistar controls and 2-month-old WAG/Rijs suggesting impaired associative memory with age (Jafarian et al., 2015).

In the GAERS model, across both low-intensity and high-intensity fear conditioning setting, GAERS showed significantly enhanced freezing response to all tones compared to the NECs, throughout all testing periods. Additionally, GAERS has also exhibited an overall delayed extinction of the conditioned fear response and increased freezing towards contextual cues compared to the NEC strain. A significant increase in the duration of freezing before and after the delivery of conditioned stimulus was reported in GAERS strain compared to the NEC strain (Marks et al., 2016b; Marks et al., 2019). This indicates exaggerated fear-related associative learning.

In contrast, CFC tests conducted on *Pcdh19* knockdown rats showed a pronounced reduction in freezing behavior upon re-exposure to stimulus 24 h after conditioning compared to the control group suggesting impaired formation or recall of associative memory (Cwetsch et al., 2022).

#### 3.4.5 Learning and adaptive memory

Learning and adaptive memory were primarily assessed using the Morris Water Maze (MWM) and Barnes Maze Test (BMT), which evaluate acquisition of spatial information across repeated trials and the ability to adjust to new task demands.

In the MWM test, non-aripiprazole-treated WAG/Rij rats displayed longer escape latencies during the acquisition phase compared to the treatment group. On day 7untreated control rats continue to exhibit prolonged escape latencies which can be ameliorated with aripiprazole treatment, highlighting a potential therapeutic effect (Russo et al., 2013).

Across multiple mouse models, including those carrying *Scn1a*, and *Gabrb3* mutations, spatial learning impairments were consistently reported. Mutant mice demonstrated a longer latency to locate the platform or target location compared to wild-type mice (Dutton et al., 2017; Gheyara et al., 2014; Lena and Mantegazza, 2019; Nwosu et al., 2023; Phillips et al., 2014; Qu et al., 2020; Qu et al., 2023; Salgueiro-Pereira et al., 2019). They also required extra training days to reach performance criteria in BMT and MWM as compared to their wild-type counterparts. Moreover, instead of utilizing target-orientated search strategies, mutant mice often relied on random search behaviors, contributing to inefficient target location arrival.

### 3.5 Secondary outcome

#### 3.5.1 ASD-related behaviors

Autism spectrum disorder (ASD)-related behaviors, including social interaction deficits and repetitive behaviors, were evaluated using paradigms such as the three-chamber social interaction test, ultrasonic vocalizations (USV), huddling, and stereotypy assays.

*Pcdh19* mutant rats exhibited significantly reduced vocalization in the USV test, pronounced isolative behavior in the huddling test, and reduced sociability index in the three-chamber test compared to control littermates. Additionally, they displayed significantly quicker response toward an acute thermal stimulus than control littermates particularly in males (Cwetsch et al., 2022). Consistent social interaction deficits were observed in other mutant strains using the three-chamber test, including *Scn1a* and *Gabrb3* models (Qu et al., 2020; Qu et al., 2023; Salgueiro-Pereira et al., 2019). Intriguingly, *Gabrb3* ^*+/N328D*^ knock-in mice did not display significant sociability deficits compared to their wild-type (Nwosu et al., 2023).

Repetitive behaviors were evaluated through self-grooming, marble burying, and OFT (Lena and Mantegazza, 2019). Young *Scn2a* mutant mice spent significantly more time burying marbles and engaging in self-grooming compared to adult *Scn2a* mutants and wild-type mice. Additionally, another study found that *Scn1a* ^*RH/+*^-SIH mutant mice displayed stereotypic behaviors in the OFT (Salgueiro-Pereira et al., 2019).

#### 3.5.2 Anxiety

Anxiety-like behavior was primarily assessed using the elevated plus maze (EPM), OFT, light-dark transition test, and looming threat paradigms, which measure rodent’s avoidance of open or aversive spaces.

Eight out of eleven studies reported significantly lower center entries and reduced exploration of the center of the arena compared to control and aripiprazole-treated groups in the OFT or EPM, indicating increased anxiety-like behavior in mutant rodents (Aguilar et al., 2018; Bender et al., 2016; Gheyara et al., 2014; Lena and Mantegazza, 2019; Qu et al., 2020; Qu et al., 2023; Russo et al., 2013; Salgueiro-Pereira et al., 2019). In non-treated WAG/Rij rats, reduced rearing and grooming behavior were also noted (Russo et al., 2013).

Mutant rodents often spent significantly less time in the open arms, exhibited reduced locomotor activity and increased freezing behavior, consistent with heightened anxiety-like responses (Gheyara et al., 2014; Marks et al., 2016b; Qu et al., 2020; Qu et al., 2023; Russo et al., 2013). These anxiety-related behaviors were reduced by aripiprazole treatment (Russo et al., 2013). OFT studies showed that mutant rodents preferred spending less time in the center zone (Dutton et al., 2017; Gheyara et al., 2014; Qu et al., 2023; Salgueiro-Pereira et al., 2019). GAERs rats made fewer open arm entries compared to NEC rats regardless of age (Marks et al., 2016b). Besides, GEPR-3s presented with a reduced number of head pokes into the open arm and higher frequency of stretch-attend posture than SD rats in the closed-arm, suggesting elevated anxiety (Aguilar et al., 2018). Additional studies revealed thatGEPR-3s strain also exhibited spent less time in the light compartment of the light-dark transition test and froze longer during the looming threat exposure (Aguilar et al., 2018). Interestingly, sex difference were noted asfemale rats made significantly more open-arm entries than male rats regardless of age and strain (Marks et al., 2016b).

Intriguingly, not all models showed heightened anxiety. There was no significant difference in the time spent in the central arena between GAERS and control littermates (Marks et al., 2016b). *Scn1a* mutants, *Gabrb3* ^*+/N328D*^ knock-in mice, and young *Scn2a* mutant mice were also revealed to exhibit normal anxiety levels in several paradigms (Dutton et al., 2017; Lena and Mantegazza, 2019; Nwosu et al., 2023).

Overall, most genetic epilepsy models exhibit heightened anxiety-like behaviors, though variability exists depending on genetic background, strain, and age. Certain pharmacological interventions, such as aripiprazole, may mitigate these effects.

#### 3.5.3 Locomotion deficits

Locomotor activity was assessed using the Open Field Test (OFT) and dedicated locomotor activity chambers, which measure exploratory behavior, rearing frequency, and distance travelled.

Mice carrying *Scn1a*, *Gabrb3* ^*+/D120N*^, and *Gabrb3* ^*+/N110D*^ mutations showed significantly increased hyperactive features such as frequency of rearing and total distance traveled compared to the wild-type littermates (Dutton et al., 2017; Gheyara et al., 2014; Qu et al., 2020; Qu et al., 2023; Salgueiro-Pereira et al., 2019).

In contrast, reduced locomotion activity was observed in *Gabrb3* ^*+/N328D*^ knock-in mice, indicating that not all GABAergic mutations produce hyperactivity (Nwosu et al., 2023).

These findings indicate that locomotor outcomes vary depending on genetic mutation, with some models demonstrating hyperactivity while others show reduced activity. Such discrepancies suggest that different genetic epilepsies may exert distinct effects on neural circuits regulating motor behavior.

#### 3.5.4 Depression

Depressive-like behaviors were evaluated using paradigms such as the Forced Swim Test (FST), Sucrose Consumption Test (SCT), and Tail Suspension Test (TST), which assess behavioral despair and anhedonia.

In the FST, *Scn1a* mutants did not show significant difference in immobility time compared to controls (Dutton et al., 2017). In contrast, WAG/Rij rats and low-dose aripiprazole-treated rats displayed increased immobility time compared to other groups, although higher dose aripiprazole treatment reduces immobility, suggesting antidepressant effect (Russo et al., 2013). A different study reported that young *Scn2a* mutants spent significantly less time being immobile compared to their controls, while immobility was observed more in adult *Scn2a* mutants, suggesting some developmental differences (Lena and Mantegazza, 2019).

The SCT revealed that GEPR-3s and untreated WAG/Rij rats demonstrated significantly reduced sucrose preference compared control or aripiprazole-treated rats, indicating anhedonic behavior (Aguilar et al., 2018; Russo et al., 2013).

Overall, depressive-like phenotypes were observed in several GE models, though results varied by genetic background and age. Importantly, pharmacological intervention with aripiprazole was able to reduce depressive behaviors in certain strains, suggesting potential therapeutic avenues.

### 3.6 Quality assessment

Overall, the quality assessment revealed that most studies demonstrated unclear risk of bias in all domains. While sequence generation and baseline characteristics were often described, reporting bias and attrition bias were rarely addressed. A summary of the SYRCLE RoB assessments for each study is presented in Table 2.

**TABLE 2:** Systematic Review Centre for Laboratory Animal Experimentation Risk of Bias (SYRCLE RoB tool) assessing quality of preclinical animal studies.

Studies	Q1	Q2	Q3	Q4	Q5	Q6	Q7	Q8	Q9	Q10	Overall
Qu et al. (2020)	U	Y	U	U	U	U	U	U	Y	U	Unclear
Gheyara et al. (2014)	U	Y	U	U	U	U	U	U	U	U	Unclear
Phillips et al. (2014)	U	Y	U	U	U	U	U	U	U	Y	Unclear
Bender et al. (2016)	U	U	U	U	U	U	U	U	Y	Y	Unclear
Russo et al. (2013)	U	U	U	U	U	U	U	U	Y	U	Unclear
Nwosu et al. (2023)	U	Y	U	U	U	U	U	U	Y	U	Unclear
Lena and Mantegazza (2019)	U	Y	U	Y	U	N	U	U	Y	U	Unclear
Jafarian et al. (2015)	U	Y	U	U	U	U	U	U	U	U	Unclear
Salgueiro-Pereira et al. (2019)	U	Y	U	U	U	U	U	U	Y	U	Unclear
Dutton et al. (2017)	U	Y	U	U	U	U	U	U	U	Y	Unclear
Cwetsch et al. (2022)	U	Y	U	U	U	U	U	U	Y	U	Unclear
Aguilar et al. (2018)	U	U	U	U	U	U	U	U	Y	U	Unclear
Qu et al. (2020)	U	Y	U	U	U	U	U	U	Y	U	Unclear
Marks et al. (2019)	U	U	U	N	U	U	U	U	Y	U	Unclear
Marks et al. (2016a)	U	Y	N	U	N	U	U	Y	U	U	Unclear
Marks et al. (2016b)	U	Y	N	U	U	U	U	Y	U	U	Unclear

Abbreviations: N, no; Y, yes; U, unclear.

Questions.

1. Was the allocation sequence adequately generated and applied?

2. Were the groups similar at baseline or were they adjusted for confounders in the analysis?

3. Was the allocation adequately concealed?

4. Were the animals randomly housed during the experiment?

5. Were the caregivers and/or investigators blinded from knowledge which intervention each animal received during the experiment?

6. Were animals selected at random for outcome assessment?

7. Was the outcome assessor blinded?

8. Were incomplete outcome data adequately addressed?

9. Are reports of the study free of selective outcome reporting?

10. Was the study apparently free of other problems that could result in high risk of bias?

## 4 Discussion

### 4.1 Cognitive dysfunction in genetic epilepsy

Two main hypotheses have been proposed to explain the cognitive dysfunction observed in genetic epilepsy models: one emphasizing seizure-related neuronal damage and the other implicating gene-driven alterations which are independent of seizure activity. According to the first hypothesis, cognitive impairment stems from persistent or recurrent seizures that lead to chronic neuronal damage and oxidative stress in brain regions responsible for memory and learning (Holmes, 2015). This was supported by studies investigating *Scn1a* mutant mice and in HCN1 (R43Q) mutants where prior induction of seizures before cognitive testing led to significant memory impairments (Dutton et al., 2017; Phillips et al., 2014; Salgueiro-Pereira et al., 2019). The second hypothesis was supported by a study where mice exposed to multiple SWDs presented with cognitive impairments which were not exhibited in their seizure-resistant R34Q counterpart (Phillips et al., 2014). The mutations in genes such as *Scn1a* and *Scn2a* directly disrupt cortical development, synaptic transmission and ion channel function, resulting in hyperexcitability in brain areas that compromise cognition independently of seizures. Consequently, prolonged neurological hyper-excitability will eventually lead to reduced neural function and adversely affect cognition over time (Staley, 2015). These two mechanisms are not mutually exclusive and may act in parallel, with genetic vulnerability shaping brain networks that are then further compromised by seizure activity.

Evidence from *Scn1a* R1648H mutant models further highlights the interplay between genetic vulnerability and seizure exposure in shaping cognitive outcomes. In these mutants, significant cognitive and behavioral impairments were observed only in rodents previously exposed to febrile seizures triggered by either hyperthermia or flurothyl, whereas mutants with the same genetic background, but without seizure exposure, did not exhibit comparable deficits (Salgueiro-Pereira et al., 2019). These findings suggest that the R1648H mutation alone is insufficient to cause cognitive and behavioral deficits but increases susceptibility to seizure-induced neuronal changes. These findings fortify the view specific genetic background may act as modifiers, amplifying the impact of seizure burden on cognition (Yi and Wei-Wei, 2023). However, it remains unclear whether this specific genetic mutation alone exacerbates the cognitive phenotype of genetic epilepsies or primarily acts through seizure-mediated mechanisms.

While several rodent models (e.g., *Scn1a*, *Scn2a*, and *Gabrb3* mutants) represent specific genetic mutations associated with epilepsy, others such as GAERS or WAG/Rij rats display spontaneous spike–wave discharges typical of GGEs. Although full validation of human syndromes remains ongoing, these models offer crucial insights into how genetic or neurophysiological disturbances contribute to cognitive dysfunction. Pharmacological studies using these models, such as Russo et al. (2013), further inform how antiepileptic interventions may modulate cognitive outcomes. WAG/Rij rats consistently demonstrated age-dependent deficits in cognitive functions such as learning, long-term, or adaptive memory. In the MWM test, an age-dependent decline in learning and memory function is observed which is consistent with earlier findings of deficits in active and passive avoidance tasks (Leo et al., 2019; Sarkisova and van Luijtelaar, 2011). These impairments in the WAG/Rij strain may be explained by an age-dependent increase in SWD frequency and duration, consistent with previous studies and may not be related to the ontogenesis of GE (Jafarian et al., 2015; Karson et al., 2012; Leo et al., 2019; Sarkisova and van Luijtelaar, 2011). This is further supported by evidences showing that Wistar rats also demonstrated poorer memory functions in cognitive tasks at older ages which is concurrently associated with the development of SWDs (van Luijtelaar et al., 1995). The cerebral hypoperfusion experienced during SWD episodes may underlie these deficits, as older WAG/Rij rats show increased neuronal apoptotic cells, dark neurons, and caspase-3 activity in the affected brain areas (Jafarian et al., 2015). These findings suggest that in WAG/Rij rats, cognitive impairment is strongly associated with seizure-induced pathology. However, future research is needed to explore whether genetic abnormalities in the WAG/Rij strain might contribute to the cognitive deficits, as opposed to solely attributing them to seizure-induced damage.

Other studies support the hypothesis that genetic mutations themselves directly drive cognitive impairment, independent of seizure burden. For instance, both young (49-day-old) and adult (200-day-old) *Gabrb3* ^*+/D120N*^ mutants exhibited poor spatial memory and learning across the BMT and MWM (Qu et al., 2020). The persistence of deficits across developmental stages suggests that the impairments arise from the underlying mutation rather than cumulative seizure effects. This pattern mirrors human syndromes such as DS, Lennox-Gastaut syndrome (LGS), and CAE, that generally manifest during the pediatric years of life and persists, regardless of seizure control (Anwar et al., 2019; Datta et al., 2023; Smith et al., 2018). Unfortunately, this poses a double whammy for those affected, as cognitive impairments are often more severe in individuals who develop epilepsy at a young age (Lenck-Santini and Scott, 2015).

Findings from *Scn1a* knockdown studies in the medial septum and diagonal band of Broca (MSDB) further illustrate how gene-specific effects can produce selective cognitive deficits. In a study by Bender et al. (2016), the knockdown of Nav1.1 in the MSDB resulted in a short-term working memory deficit in the mutant rats evidenced by the poor path efficiency in MWM and poor choice accuracy in the T-maze rewarded alternation test (Bender et al., 2016). Electrophysiological data revealed selective and targeted damage to the burst-firing GABAergic neurons, as well as loss of hippocampal theta oscillations, both of which are critical for spatial working memory (Bender et al., 2012). These findings align with prior studies using neurotoxins to selectively damage MSDB neurons which similarly resulted inworking memory but not reference memory deficits (Dwyer et al., 2007; Pang et al., 2011). Interestingly, Nav1.1 knockdown secondary to *Scn1a* mutation did not impair spatial reference memory, highlighting the task-specific nature of these effects (Leo et al., 2019). Together, these results suggest that functional GABAergic impairments due to *SCN1A* mutations disrupt hippocampal theta frequency, selective working memory impairments and not the reference memory.

Mutations in GABAA receptor subunits, which underlie syndromes such as Lennox-Gastaut syndrome (LGS) and infantile spasms syndrome (ISS), consistently produce severe cognitive impairments in rodent models. LGS models carrying *Gabrb3* ^*+/D120N*^ and *Gabrb3* ^*+/N328D*^ mutations as well as infantile spasms syndrome (ISS) models carrying *Gabrb3* ^*+/N110D*^ mutations exhibited similar deficits in learning and memory, suggesting a cognitive deterioration phenotype typically seen in epileptic encephalopathies and progression of these genetic epilepsies (Nwosu et al., 2023; Qu et al., 2020; Qu et al., 2023). Clinically, cognitive impairment affects more than three-quarter of LGS patients, while 70%–90% of ISS patients develop intellectual disability (Zupanc, 2003). Notably, aberrant interictal epileptiform activity which interfere with cerebral function and normal brain developmental can still cause cognitive impairment even when the seizures are controlled (Aldenkamp and Arends, 2004; Stroink et al., 1998). This mechanism may explains the neurocognitive and behavioral deficits in *Gabrb3* ^*+/N110D*^ mutants whose interictal brain waves differed significantly from wild-type controls, suggesting that abnormal background brain rhythms contribute to progressive cognitive regression in these models (Qu et al., 2023).

GABAergic neurons, distributed throughout multiple brain regions, play a critical role in maintaining inhibitory balance and cognitive stability. In *Gabrb3* ^*+/N328D*^ mutated mice, reduced expression of *Gabrb3* was observed in the thalamus, cerebellum, and hippocampus regions, postulating a decrease in the stability of the mutant β3 subunit protein (Nwosu et al., 2023). Given the hippocampus’s well-established role in memory formation and retrieval, deficits in GABAergic inhibition within the hippocampus may explain the impaired memory and learning ability observed in *Gabrb3* ^*+/N328D*^ mutants. Notably, β3 subunits are highly expressed in the adult hippocampus, reinforcing their importance for normal cognitive processing (Hörtnagl et al., 2013).

Recognition memory allows animals to distinguish familiar from novel stimuli and has been linked to perirhinal cortex (Moreno-Castilla et al., 2018). In drug-naive GAERS, CMOR memory deficits were observed without aversive stimuli, minimizing the influence of anxiety on performance (Marks et al., 2016a). These impairments are likely linked to the dysfunction of perirhinal cortex, a region implicated in visual recognition memory (Winters and Reid, 2010). GAERS are reported to exhibit altered perirhinal cortex function, likely due to the mutation in CaV3.2 T-type calcium channels (Powell et al., 2009). This is notable given the high density of T-type calcium channel the perirhinal cortex (Talley et al., 2000). Hence, recognition memory impairments in GAERS may be mediated perirhinal cortex dysfunction linked to altered T-type calcium channel activity.

Not all studies have reported consistent recognition memory impairments, and several confounding factors may influence outcomes. In GEPR-3s rats, apparent memory consolidation deficits may be confounded by heightened anxiety-related behaviors (Aguilar et al., 2018). Elevated corticosterone levels in stressful settings can motivate stress-induced novelty avoidance behavior and impair NORT performance, without reflecting true memory deficits (Vargas-López et al., 2015). Consistently, elevated corticosterone levels have been reported in GEPR-3s rats, which may underlie their poor performance in recognition tasks (Aguilar et al., 2018). In contrast, young *Scn2a* mutant mice displayed age-dependent memory impairment which did not last till adulthood (Lena and Mantegazza, 2019). This developmental pattern may reflect the predominance of NaV1.2 voltage-gated sodium channels in the hippocampus during the initial 3 weeks of life, which is later taken over by Nav1.6 sodium channel subtype during maturation (Boiko et al., 2003). These findings underscore that recognition memory outcomes may be shaped by extrinsic confounds, such as stress and anxiety, and intrinsic developmental factors, such as ion channel maturation. Moreover, given the frequent comorbidity of ADHD with ASD, attentional dysfunction may also contribute to recognition impairments in young Scn2a mutants.

Associative memory outcomes varied across various GE rat strains. In WAG/Rij rats, deficit in associative memory is likely due to an age-dependent increase in seizure frequency and duration resulting in neuronal disruptions (Jafarian et al., 2015; Karson et al., 2012; Leo et al., 2019). In contrast, *Pcdh19* mutant rats lacked significant seizure history but showed noticeable impairments in associative memory during the CFC test. Supporting this, it was reported that *Pcdh19* heterozygous female mice demonstrated consistent reduced fear response in fear conditioning tests, while hemizygous male mutants showed no alterations in fear response (Hayashi et al., 2017; Hoshina et al., 2021). *Pcdh19*-transfected rats also demonstrated poor long-term memory functions in the NORT. Together, these findings suggest that the associative memory impairments in WAG/Rij rats are likely seizure-driven, whereas in *Pcdh19* mutants, they may arise directly from the underlying genetic defect.

In contrast to WAG/Rij and *Pcdh19* models, GAERS models exhibited enhanced associative learning, with stronger cued and contextual memory in response to fear (Marks et al., 2016b; Marks et al., 2019). Similar enhancements were reported in aversive learning tests such as the two-way active avoidance tests (Getova et al., 1997; Sitnikova, 2024). However, these improvements may be confounded by the delayed extinction of conditioned fear, raising the question of whether the results were due to dysfunctional inhibitory learning, augmented fear conditioning, or heightened anxiety behavior (Marks et al., 2016b; Marks et al., 2019). Thus, the disparity in associative cognition observed across strains highlights both test-dependent variability and the likelihood that different mechanisms such as seizure burden, genetic mutation, or altered emotionality, underlie associative memory outcomes in GE models.

These findings also highlight how genetic mutations in epilepsy can influence not only cognition but also broader behavioral domains. The next section considers how GE models recapitulate behavioral phenotypes, including autism spectrum disorder (ASD)-related traits, anxiety, and depression.

### 4.2 Behavioral deficits in genetic epilepsy

Mutant rodents frequently exhibited behavioral phenotypes resembling autism spectrum disorder (ASD), including repetitive behaviors, deficits in social interaction and sensory hypersensitivity. For example, *Pcdh19* transfected rats demonstrated socio-behavioral impairments from early life that persisted into adulthood as well as sensory hypersensitivity which are consistent with ASD-like traits (Cwetsch et al., 2022). Heterozygous *Pcdh19* knockout female mice also showed reduced sociability in the three-chamber test, resembling autism-like behavior (Lim et al., 2019). Interestingly, sex difference have been noted, with male rodents exhibiting greater pain hypersensitivity compared to females (Hayashi et al., 2017). Similarly, young *Scn2a* mutant mice displayed repetitive behaviors and impaired social interaction, likely reflecting dysfunctions in cortico- striatal circuits in the brain that regulate social and repetitive behaviors (Lena and Mantegazza, 2019). These findings suggest that genetic epilepsies involving *Pcdh19* and *Scn2a* mutations may directly contribute to ASD-like phenotypes. Across multiple studies, anxiety-like behavior were consistently reported in rodent GE models. Two-month-old WAG/Rij rats without seizure history demonstrated increased anxiety behavior and stress reactivity compared to age-matched Wistar rats, suggesting a strain effect rather than seizure-induced pathology (Fedosova et al., 2015). GAERS model similarly exhibited heightened anxiety behavior in the EPM (Jones et al., 2008; Powell et al., 2014). However, results from the OFT appeared to be task-specific and age-dependent (Marks et al., 2016b). GEPR-3s consistently demonstrated anxiety-like behavior across all paradigms, which were attributed to serotonin abnormalities in the brainstem (Aguilar et al., 2018; Dailey et al., 1992). *Scn1a* mutants displayed modest increase in anxiety, consistent with Nav1.1 haploinsufficiency models that increased thigmotaxis in the OF test (Ito et al., 2013). Collectively, these findings indicate that genetic background and neurotransmitter abnormalities may modulate anxiety phenotypes across GE models.

The *Scn2a* model revealed a clear developmental contrast in anxiety phenotypes. Young *Scn2a* mutants demonstrated lower anxiety levels in EPM and OFT, as well as reduced immobility during the TST, in contrast to adult mutants (Lena and Mantegazza, 2019). Comparable behavioral findings have been reported in BTBR mice, where reduced anxiety-like behavior was also observed (Pobbe et al., 2011; Silverman et al., 2010). Researchers hypothesized that such behavioral findings were most likely explained by modifications of the hypothalamic-pituitary-adrenal (HPA) axis. Supporting this, reduced NaV1.2 expression in excitatory pathways of amygdala, hypothalamic, and limbic circuits have been shown to modify the HPA axis (Ogiwara et al., 2018). During early development, the amygdala primarily regulated emotional response towards aversive stimuli, but later phases of development the role is then taken over by the prefrontal cortex which exerts a top-down regulation and enhances emotional control (Gee et al., 2013). Consequently, reduced signaling from the amygdala may attenuate HPA activity during stress, explaining the lower anxiety phenotypes observed in young *Scn2a* mutants.

Evidence for depressive-like behaviors was also reported across GE models, though some findings may reflect confounding by anxiety. For instance, GEPR-3s exhibited reduced sucrose preference in the SCT, though this may reflect novelty-suppressed feeding due to heightened anxiety rather than depression (Aguilar et al., 2018; Bodnoff et al., 1988). Supporting this, fluoxetine, an anxiolytic agent, restored feeding behaviors in a corticosterone-induced anxiety model (Mendez-David et al., 2017). In a tail suspension test, adult*Scn2a* mutant mice, showed increased immobility, suggestive of depressive-like symptoms (Lena and Mantegazza, 2019). Similarly, WAG/Rij rats at six and 12 months old also demonstrated increased immobility time in the FST (Leo et al., 2019). Collectively, these findings indicate that depressive-like behaviors may emerge in GE models, although careful interpretation is warranted due to overlap with anxiety-related outcomes.

Some findings diverged across models, highlighting the importance of genetic background. For example, *Gabrb3* ^*+/N328D*^ mutants showed reduced locomotor activity, impaired social interaction and anxiety symptoms, whereas *Gabrb3* ^*+/D120N*^ mutants did not, suggesting that motor deficits may secondarily influence behavioral outcomes (Nwosu et al., 2023). This observation highlights that even within the same syndrome, distinct mutations can result in divergent behavioral outcomes, reflecting the heterogeneity of GE models.

Several limitations should be acknowledged when interpreting the findings of this review. Genetic epilepsies are highly heterogeneous, both in their underlying pathophysiology and in the cognitive and behavioral phenotypes observed in animal models. As such, the outcomes summarized here are mutation- and syndrome-specific and should not be generalized across all forms of GE. In addition, some of the included studies incorporated treatment interventions; however, these were only considered when baseline cognitive or behavioral outcomes in untreated mutant animals were available, ensuring that the synthesis focused on the intrinsic phenotypes of GE models rather than treatment efficacy. We also recognize that our search strategy, which focused on the broad term “genetic epilepsy,” did not capture every gene- or syndrome-specific model described in the literature. Given the extensive and heterogeneous range of genes implicated in GE, it was not feasible to include all of them at this stage. Nonetheless, we believe that our approach was sufficient to provide a representative overview of cognitive impairments in rodent models of GE. Furthermore, the quality appraisal using the SYRCLE Risk of Bias tool highlighted that most included studies were rated as having an overall “unclear” risk of bias. Key methodological aspects such as sequence generation, allocation concealment, random housing, and blinding of investigators or outcome assessors were seldom reported. Even in more recent studies, including those assessing GAERS and pharmacological interventions, details on randomization and blinding were often absent or ambiguous. Therefore, while our review synthesizes available findings on cognition in genetic epilepsy models, the interpretability and generalizability of these results remain constrained by methodological shortcomings in the primary studies. Together, these considerations highlight the complexity of drawing broad translational conclusions and emphasize the importance of syndrome-specific and methodologically rigorous future studies.

## 5 Conclusion

This systematic review comprehensively examine the cognitive and behavioral impairments across rodent models of GE, highlighting both common features and syndrome-specific differences. While many models effectively recapitulate deficits observed in patients, outcomes vary depending on mutation, strain, and age, reflecting the heterogeneity of GE. Our quality assessment revealed significant reporting gaps, with most studies rated as having an “unclear” risk of bias, underscoring the need for more rigorous and transparent experimental designs. Despite these limitations, rodent models remain indispensable for studying seizure-related and gene-driven contributions to cognitive decline. This review provides a foundation for future syndrome-specific studies and encourages the use of diverse animal models to better elucidate the mechanisms underlying cognitive comorbidities in genetic epilepsy.

## Data Availability

The original contributions presented in the study are included in the article/supplementary material, further inquiries can be directed to the corresponding author.
